# Discolored Structure of Devitalized Teeth—Causes, Method of Treatment and Agents Employed

**Published:** 1897-01

**Authors:** H. T. Harvey

**Affiliations:** Battle Creek, Mich.


### Discolored Structure of Devitalized Teeth--Causes, Method of Treatment and Agents Employed.

BY H. T. HARVEY, D.D.S., BATTLE CREEK, MICH.

Read before the Michigan State Society.

My thirty minutes' paper will be confined to the "Causes of Discolored Tooth-Structure," or devitalized teeth, the method and agents employed, and results obtained by myself in the bleaching of the same. The demands upon the progressive practitioner of to-day, from the patients for relief from this class of teeth, is constantly becoming more frequent.

How often do we have a patient, upon occupying our operating chair, ask, "Doctor, can you not do something to restore the natural color of that tooth? I would prefer crowning or extraction rather than endure the painful sight of it longer." And how often do we see a beautiful set of teeth marred and disfigured by a discolored member invariably located in the most conspicuous part of the mouth.

My first consideration of this subject will be to partially, at least, define the causes of "Discolored Tooth-Structure." We are all well aware that the commencement of discoloration occurs from a lack of life's nourishment, which always results in the death of the pulp, and in the majority of cases putrescent pulp occurs. Its action upon the nerve fibrilla of the canaliculi is, as yet, a phenomena as to the actual agent that discolors the organic part of the dentine. Some authorities have advanced the theory that acid was the destructive agent. I can not but concede that acid is the indirect cause in a majority of cases, inasmuch as it has been shown to the profession by scientists that acids decay tooth-structure, and decayed tooth-structure is the primary cause of putrescent pulp to a large extent, yet, I contend that in cases where a sound tooth has received an injury or shock from violent means, or otherwise, there can not exist acid in the pulp canal or in the canaliculi unless there is a generative action in the pus of the putrescent pulp. In some cases we find the pulp dried up and in these cases the staphylococcus pyogenus aureus which did exist were starved earlier, and in this class of teeth we do not find the discoloration of the structure of the tooth so intensely dark.

In the chemical action of the pus acid is formed which produces the acid condition. We have excellent authorities who affirm the existence of acid in all pus. We have others, of equal renown, who claim there is no chemical action of the organisms of the dentinal tubuli, but that after the death of the pulp, which necessarily extends the same fate to the live organisms of the canaliculi, and that there is always discoloration of live matter when it succumbs to disease and death, that it merely lies in that discolored condition dormant, and that the structure of the dentine and enamel being semi-transparent or translucent, the darkened color of the tooth is caused by refracted light, and that at no time is the interstructure between the dentinal tubuli discolored.

I find, however, that the micrococci fetidus germs, which exist in the decayed tooth-structure, and pus germs are prevalent in large quantities, from my limited observation and study of this phase of the subject. We have a class of germs to deal with originating from the pus called staphylococcus pyogenus aureus. These germs obtain entrance into the pulp canal one of three ways: through a decayed cavity, the micrococci fetidus being transformed into the staphylococcus pyogenus aureus; or through the arterial supply of the blood of the pulp; or through the circulating medium, the peridental membrane and cementum, through the latter medium, it is claimed, the staphylococcus pyogenus aureus can gain admission through absorption. I hold to the theory, however, that the staphylococcus pyogenus inoculates itself into the pulp, thence into the organisms of the canaliculi through the blood supply.

Inasmuch as these pus germs are very prevalent in the blood the staphylococcus pyogenus aureus is inactive only until they find tissues succumbing to the disease.

I am thoroughly convinced that there is a limit to the life of the micrococci fetidus, and the staphylococci pyogenus aureus, as it is found in the tooth-structure. Their source of supply of nourishment is eliminated from the tissues of the pulp, and when that organ ceases to receive blood supply from its natural source, and its only life supply left is what it may obtain from the arterial supply of the peridental membrane and cementum, which necessarily ends the nefarious work of these germs whose life is cut off, and ultimately starved for the want of nourishment.

My mode of procedure is to first excavate the tooth from all decay, apply the rubber-dam to the tooth to be bleached, also to the ones approximating it upon either side. My object being to get the shade of the adjoining teeth, using them as a guide for the requisite color to be attained, and to exclude all moisture of the mouth and render the cavity more accessible. I covered the approximating surface of the adjoining teeth, together with the tooth to be bleached, with a very thin coat of vaseline or white paraffine wax, which is transparent; I then cleanse the cavity with a 95-percent solution of alcohol, having previously cleansed the root-canal and rendered it antiseptic, then apply aqua ammonia, and dry thoroughly, using a hot-air syringe, which proves most valuable in the vaporization of all moisture of the teeth. I then take a small platinum broach with a pledget of cotton or bibulous paper twisted thereon, and thoroughly saturate the tooth with 25-percent ethereal solution of pyrozone, allowing the greatest quantity to remain in the tooth possible. I then turn the hot-air blast upon the tooth, which rapidly vaporizes the pyrozone and causes it to permeate the canaliculi. This procedure is repeated until the tooth-structure assumes its requisite whiteness or color. Following the experiment of Drs. Westlake and W. J. Morton, as described in the *International Dental Journal* and *Dental Cosmos* for 1895, I tried the cataphoric applications with the pyrozone, using 25-percent ethereal solution, and found, with the galvanic current, I could attain the same results as with the hot air, and in about one-half the time.

I now use cataphoric applications quite generally in bleaching of teeth, believing I am able to even permeate with the pyrozone, aided with the cataphoric applications, to a greater depth than with hot air.

As pyrozone is an ethereal solution, the ether permeates the canaliculi and absorbs, or thoroughly transforms, the protoplasmatic matter, and kills all germs therein.

At this stage the canaliculi and its contents must receive careful attention to obtain permanent results, and prevent the recurrence of the discoloration of the tooth. I now apply to the cavity a sufficient quantity of white shellac—using a very thin mixture—and evaporate with the hot-air blast or by using the cataphoric application; I can get the same satisfactory results as when using the galvanic current in the penetration of pyrozone. The alcoholic solution of shellac, penetrating, by capillary attraction, to a greater extent through the tooth-structure renders it air- and moisture-tight. I now apply a thin lining of oxyphosphate of zinc to prevent the appearance of gold or other filling material through the enamel, which oftentimes occurs, and which, in itself, proves very unsightly. I then fill the tooth in the ordinary manner. I have bleached one or two teeth without disturbing the nerve-canal filling, but deem it proper to remove the same in every case to render one's efforts successful. I have tried bleaching teeth with sodium peroxide, chlorate of lime, aromatic sulphuric acid, diluted, and pyrozone.

The sodium peroxide does not accomplish the desired effect, the chlorate of lime is superficial in its action, not possessing the osmosis qualities of ether as pyrozone does. Aromatic sulphuric acid is detrimental to tooth-structure, which renders it unfit for use.

Pyrozone's chemical consistency is $H_2$, $O_2$. in ether; it is mildly caustic, not to the extent of destruction of tooth-structure, yet sufficient for decided action; it may be applied to the skin without injury thereto, and I have not perceived any ill-effects from its powerful action upon the structure of the teeth.

When Messrs. McKesson & Robbins first placed pyrozone upon the market two years or more ago, I bleached several teeth with it. I had the pleasure of seeing one case some two months ago—a lady from Vicksburg, Mississippi—with a right superior central incisor-tooth badly discolored; it required about 50 minutes to bleach it, and it has retained its color remarkably well.

My modus operandi was somewhat different at that time, however, than at present, as outlined in this paper. I have practiced the bleaching teeth to quite an extent of late, and the results have been perfectly satisfactory in every case up to date. And I have every confidence that they will retain their color, as bleached.

The minimum amount of time required by me to bleach a tooth, a lateral incisor, inky-black in color, which tooth I bleached in ten minutes to the requisite whiteness; in fact, one shade whiter, allowing for retrocedence.

The manufacturers have made a great improvement in pyrozone in the last two years, and now produce a medicinal aqueous solution, together with a 5-percent and 25-percent ethereal solution. The 5-percent ethereal solution can be used for bleaching purposes, but it requires much more time; one can evaporate a 25-percent solution to any solution, if they do not desire as strong a solution as the 25 percent.

The pyrozone comes in sealed glass-tubes, labeled, giving the exact date upon which it should be used.

I offer this paper with the hope that it may induce more of my colleagues to widen their scope of practice, and give to their patients the relief that can be afforded them.

DISCUSSION.

Dr. Blackmarr, of Jackson: I wonder if it would not be well to have Dr. Harvey, or some one who has used pyrozone, to tell those who have never opened these tubes of pyrozone, how best to get the pyrozone after they have opened them, and how to do it without exploding, as I have seen done after trying to get a little pyrozone.

Dr. Harvey: Dr. Blackmarr's experience has certainly been sad in this respect—he has not been able yet to get a drop of pyrozone; I have never had the least trouble yet to open one of these tubes, and I have never yet placed one upon ice, yet those are the directions—that they should always be kept in a cool place, and after opening I place in a colored-glass bottle.

Dr. Morse: How do you place it in the dark-colored bottle from the tube?

Dr. Harvey: I pour it in.

Dr. Morse: I never had a chance to.

Dr. Harvey: That is queer! What does it do, evaporate?

Dr. Morse: I never had a chance to pour it out; it usually shot out.

Dr. Harvey: I have never had that experience. I have opened over thirty tubes, and I use a tube of pyrozone the second time for bleaching teeth, after I have opened it; and then I do not throw that away. I have found it very valuable in practice and in the treatment of ulcerated teeth; it is much superior to peroxide of hydrogen in its action on pus, and not only that, it has the same qualities as sulphuric acid—the same qualities exactly. One gets the results of both of those agents in a 25-percent solution of pyrozone, and my experience has been eminently satisfactory. I take these tubes and put the point on an anvil, and with a hammer I knock the end off, and pour it into a bottle, and have never yet broken one.

Dr. Morse: I have followed directions, and I can usually save, perhaps, a quarter of it.

Dr. Harvey: That is all you need; there is enough pyrozone in one of those tubes to bleach twenty teeth.

Dr. Morse: But it is a valuable thing to have, and why not save the whole of it? I believe there is a personal element which accounts for many of these accidents.

Dr. Ray, St. Joseph: I have had considerable experience with pyrozone since the World's Dental Congress. When the clinic was brought to my observation I took a good deal of interest in it, and after coming home I experimented quite a little, and my experiments from that time to this have been more satisfactory all the time. But I have had some trouble, like the rest of you, in opening those tubes of pyrozone. I have had no trouble except in one case, however, I lost one. I don't know where it went to; I could not detect it in the air, nor could I find any pieces, and I did not get any in my face. My mode of operating is according to directions, a good deal, in opening these tubes. I first put the tube in a pitcher of cold water and let it remain a long while; I am in no hurry about opening; I want it to get cold, and I have a towel which I have just as cold as the water and I wrap it around the tube and just expose the bare point of it, so, in case of any accident, there will not be any danger of getting the glass in the face. That is the danger I have experienced from it. I have never had any trouble only with one tube.

My practice has been in a tooth that has been discolored, and I think pyrozone will only operate upon a vegetable or animal substance. My experience in getting discoloration out of an old amalgam filling, which might have come under your observation, is that I never can remove it; in fact, the chemists themselves who manufacture it claim it will not take away a metal discoloration, and I have found that to be the case. There is, once in awhile, a case where there is an amalgam discoloration, where they have had to put it in to save the tooth in patients that were not accessible to a good dentist, and generally you can remove that with your excavator, so that you can get a very good-looking tooth. In fact, it would be a great improvement—anything would be an improvement over an old amalgam filling in a front tooth.

My way of proceeding is to thoroughly clean out the tooth, remove all of the soft matter of the dentine. I have always treated those teeth and got them first in a healthy condition before I bleach them. I remove the decay and open the roots nicely—probably up two-thirds of the way—making it perfectly safe above the gum, so that no discoloration would ever follow afterward from that, and I do not believe it will where it is thoroughly bleached out and the tooth is properly filled with cement. I do not believe it can. I generally commence with a three percent solution of pyrozone, because I think the caustic effects of five or twenty-five percent would hinder a little the action. The three percent is an acrid solution, and I like it better on that account. I commence with that and follow it up with a five percent and end up with a twenty-five percent solution. When I first commenced to use it, I used a cold blast, having no other accessible, and I realized after awhile that I could produce better results from it by having, at least, the temperature of the tooth, or even warmer than that, and now I get as much heat as the patient can stand, and produce a great deal quicker results.

It has been my habit also to protect the tooth only that I am bleaching; it would be a good thing, however, to have one or more coming through, provided you can protect the gums; it is quite unpleasant to the patient to have any of it get around the teeth and around the gums. I have found that by simply using it over one tooth—and I think, with care, you can use it over more—there would be an advantage, that is, you can ascertain exactly the color of the other tooth by having it exposed to view. My habit has been in deciding as to the color to select a tooth about the natural color near the one I have been operating on, and in that way I have brought about the desired results. When I have found about the right color, if the patient is able to stand it long enough, I would keep on until I would bleach the tooth the same day; if he were not, and I would tire him out, I would seal the cavity up with a twenty-five percent solution with gutta-percha and let it remain until the next day or the day following; in two or three instances when they have come back I have found the tooth was all right and did not need any more attention, while it was not satisfactory when I left it. I immediately fill the teeth with the cement as near the color of teeth as I can; I believe that cement is the best filling to follow up the bleaching; I believe it more thoroughly seals up those tubuli than anything else you can use to preserve the color. I think in some cases where you simply have the enamel left, as you do in many cases, that the simple yellow discoloration in a tooth would be an objection, as those do not exactly correspond with the other teeth, and that is what you want to do to have it satisfactory, and I would fill them up with simple cement and would leave them alone for quite a while—several months if it is necessary—and then remove some of that cement and fill with gold.

Now then it has been, in my hands, the only method satisfactory in bleaching teeth. I used to try it years ago, and it took me weeks and months; and when I got through with a tooth it did not have the color at all with any of the substances that were recommended for that purpose. I got tired of it and threw it aside; I would rather have taken off an old black tooth and put on a good crown, than to have seen the tooth there, and I am glad to find something that we can save that good natural tooth with ; I believe it is better than the crown.

Dr. Harvey: I will say that it is time uselessly spent to try to bleach a mineral or metallic stain out of any tooth-structure—you can not do it. I remember when I first began to practice; I had a good many approximal contour fillings, and I used some nickel Howe-posts, and in about six months we got one of the worst stains I ever saw. It penetrated every particle of that tooth structure; one of these patients came back to me after I had been using pyrozone, but I could not phase it—I wish could, I would have given a good deal.

Dr. Myers: I would like to inquire what effect pyrozone would have on a stain caused by oil of cinnamon or some of those stains where that had been used as a disinfectant.

Dr. Harvey: I will say that I have never had occasion to apply it in that manner or for that purpose. I can not see, though, why it should not remove it.

Dr. Ray: I will say the same—I have never had occasion to bleach a tooth of that kind, although I have seen one or two cases, but I have never had the pleasure of bleaching them.

Dr. Blackmarr: I think it is very fortunate that these two papers have been read together, one following the other, because they seem to be right in line—a person needs to use electricity in both cases; and, as one of our dental firms say in one of their advertisements, "Nothing succeeds like success." I think nice points have been brought out. In the first place, that pyrozone is what we want to use to bleach the teeth, and then the next thing is, what kind of teeth will it bleach. Some of us might have gone home and expected to have a mineral stain on a tooth bleached, and wondered why it would not; Dr. Harvey has explained that, and the teeth we expect to have the success on of course are those that come from organic stains, and with a subject like this there are many difficulties; for instance, the electrical current; that must be done very accurately. And the next thing is to get the pyrozone. That must be done very nicely, in order to get it, and all those things require a good deal of accuracy, and there are a great many dentists here who would go home and try it, and unless they are very accurate and understand that they must be accurate, will not have good results.

DR. S. M. WHITE, of Benton Harbor: Dr. Ray, of St. Joseph, has given you the main points on bleaching, and if I get stuck on a tooth I just step over to Dr. Ray's office and have him help me out. As for opening the tubes: When I open a tube I am sure it is going up any way, because I have an arrangement that I can drop it into, a place, and knock that top off, as spoken of by Dr. Harvey, and whatever gas goes up it does not go into my face or anybody else's face.

I want to touch upon the first paper by Dr. Morse. The paper has been very interesting to me. Of course it is a fact that the coming dentist is going to be the man that can perform operations upon these delicate tissues with the least pain, and it is a fact that the effects of cocaine on the dentine of the tooth. I do not think that will be disputed, and I believe it is a fact that the effects of electricity upon a live tooth properly administered, that is, administered very slow at first, and then the same as they apply general electrical currents on a person, starting in generally with a small current, very small, and increasing it, and by this means it does not affect the person—and I do not believe it would affect the tooth. A strong current thrown into a tooth at once might set up a shock and paralyze the nerve and set up an irritation, but I believe by using it properly there would be no ill-effects from the use of electricity upon a live tooth.

DR. J. C. PARKER: There is one precaution that I think is well enough to be taken into consideration in regard to the use of cocaine and electricity; it has been brought about by the use of it in other operations. During the last year a case occurred in Chicago where it was being used in that way. The operator put one of the electrodes at the back of the neck and used the cocaine, with the result that the woman died in the office, and it was decided that it being in direct application so near the medulla, that it was carried there and death was the result, so that it might be well to be warned in that respect, as to where you put the opposite electrode.

Dr. Metcalf: I don't know as I can occupy much time here without creating a breeze, and I don't know that we need any disturbance this morning, I don't know that we have all recovered from the one we had last evening. During the reading of these papers some one very kindly invited me to enter into a discussion of the papers, and I had to confess that really I did not know the difference between pyrozone and kerosene. I have not kept up to the times at all; I am a sort of a "left-over," a way-back dentist. But in connection with this subject there is a question that I would like to ask just for information, and I suppose all of you can answer it very readily and will be astonished at my ignorance in asking such a question; I don't know how that is, but still I am willing to confess ignorance. And the question that I would ask is this: What is it that produces discoloration in a dead tooth? We all know, of course, what produces the discoloration in a tooth that has been filled with mercurial preparations; but when we find a dead tooth, what is it that produces that discoloration? Now I would like to have some one answer that question, and I presume you will answer, all it at once, and I want to know what it is. Then, if we know what it is, we can discuss materials for bleaching intelligently. Perhaps something has been found that will restore the natural color to a tooth without ever finding out what it is that discolors, but I think it is a good thing to know what it is that discolors the tooth.

Dr. Harvey: I expected "Father" Metcalf to propound some question that would puzzle the natives, and I would like to answer his question by saying that in a case where excessive cigarette-smoking has been indulged, or that has been the practice, and the pulp has been devitalized through it, there is a stain, we know what has caused that—it is primarily through irritation, that is through either acid or an agent irritating enough to cause the death of the pulp.

Dr. Metcalf: Now, I do not believe very much in this germ theory when it comes to the discoloration of the teeth. I have always believed, though perhaps wrong in it, that the discoloration in a tooth—that we generally meet with and which we want to remove—is caused by some element in the blood that gets into the circulation through the canaliculi of the tooth-structure. Of course, we have got to work down pretty fine if we can find any iron in the substance that circulates through this canaliculi of the tooth, but that there is a circulation there of some sort I guess no one will deny, and if there is a circulation there, isn't it related to some extent to the blood, and if so, is there not iron in that?

Now, in bleaching teeth, it has always been my practice to use some material that would have a chemical action on iron. I have not always succeeded in bleaching teeth; I have done fairly well sometimes, but I apprehend you are doing much better now than I have ever done; I have had to proceed on not as intellilines as you do nowadays. But I think we should know what it is that produces the discoloration. Of course, I can see, possibly, that in smoking cigarettes the nicotine or the oil from the tobacco, whatever it is, may possibly get in the tooth in some way or other and discolor it, but I do not think that there are any germs there; I think that nicotine would knock the germs higher than Gilderoy's kite.

Dr. Parker: In regard to this matter of discoloration, we very often find that a tooth, after it has had a rubber dam on it for an hour, when taken off and compared with the other teeth is very much lighter, and remains so for quite a while, two or three hours afterward. Now, what has taken place and what is the result when the color is restored?

Dr. Ray: I am not a very thorough chemist, but in the application of pyrozone I made up my mind to this fact—whether in the right or not—that the fiber of the odontoblasts that occupy gent the tubuli of the tooth or the nerve pulp-chamber become dead, and decomposition sets in there and that turns dark; it must necessarily follow that the fiber in those tubuli will naturally turn dark, in time. Now, the question is, how do you change that? The nature of the ethereal solution of pyrozone will change that from a dark to a light color, and then what do you want? Do you want something to remove that from those tubuli? You want something to remove that from the tubuli and you want something powerful enough, that will reach the terminus of this tubuli, and that is at the border of the enamel, and I have found in pyrozone something more effective than anything I have ever tried in that direction.

Dr. Harvey: I will say, in answer to Dr. Metcalf, that it is conceded by a large majority of the eminent physicians of this country that at all times the blood has germs in it, and that those germs lie dormant and attack the weakest point of the body when that point succumbs to disease. Now, that is an acknowledged fact. Consequently, through the circulation of the blood those germs, when disease attacks the pulp of a tooth, can enter through the circulating medium and do their destructive work just as well as they can when there is a breaking down in any other tissue of the body.

If you, gentlemen, will go at it right and go to bleaching teeth with pyrozone, yon will never regret it as long as you live. The results are more than satisfactory, and you will get these testimonials from all over the country. I have never heard of a man who has tried the use of pyrozone, and who has used it correctly, but what has been greatly pleased with it.

It was moved and supported that some appropriation be made for getting out circulars and application blanks, including an invitation, to send out to the different dentists throughout the State that are eligible to membership in the association, inviting them to become members of the association and attend the meetings, which motion was carried.

The meeting here adjourned.

